# Weight- perception in male career firefighters and its association with cardiovascular risk factors

**DOI:** 10.1186/1471-2458-12-480

**Published:** 2012-06-25

**Authors:** Dorothee M Baur, Costas A Christophi, Antonios J Tsismenakis, Sara A Jahnke, Stefanos N Kales

**Affiliations:** 1 Department of Environmental Health, Environmental and Occupational Medicine and Epidemiology (EOME), Harvard School of Public Health, Boston, MA, USA; 2 The Cambridge Health Alliance, Harvard Medical School, Employee Health and Industrial Medicine, Cambridge, MA, USA; 3 Cyprus International Institute for Environmental and Public Health in association with Harvard School of Public Health, Cyprus University of Technology, Limassol, Cyprus; 4 Boston University School of Medicine, Boston, MA, 02118, USA; 5 The Institute for Biobehavioral Health Research, National Development and Research Institutes, Leawood, KS, USA; 6 The Cambridge Health Alliance, 1493 Cambridge Street. Macht 427, Cambridge, MA, 02139, USA

**Keywords:** Weight perception, Cardio-respiratory fitness, Firefighters, Obesity, Cardiovascular-risk factors

## Abstract

**Background:**

The prevalence of obesity has reached epidemic proportions worldwide, and is also increasing among public safety professionals like firefighters who are expected to be fit and more active. The present study evaluates the associations among Body Mass Index (BMI), weight perception and cardiovascular risk factors in 768 male career firefighters from two Midwestern states in the United States.

**Methods:**

A physical examination was performed and fasting blood samples were taken. Cardio-respiratory fitness (CRF) was determined from symptom- limited maximal treadmill exercise testing with electrocardiogram (ECG) monitoring and estimation of oxygen consumption (metabolic equivalents, METS) using the Bruce protocol. A health and lifestyle questionnaire was administered with standardized written instructions for completion. Self-reports of weight perception were extracted from responses to the completed multiple choice questionnaire. Baseline characteristics were described using the mean (standard deviation) for continuous variables and frequency for categorical variables. Group comparisons were calculated using analysis of variance (ANOVA). Linear models and logistic regression models were used to adjust for possible confounders. Logistic regression analyses were used to calculate the odds ratios of underestimating one’s weight category.

**Results:**

A high proportion of overweight and obese male career firefighters underestimate their weight categories (68%). The risk of underestimating one’s weight category increased by 24% with each additional unit of increasing BMI after adjustment for age and CRF. When divided into six groups based on combinations of measured BMI category and weight perception, there were significant differences among the groups for most cardiovascular risk factors. After adjustment for age and BMI, these differences remained statistically significant for CRF, amount of weekly exercise, prevalence of Metabolic Syndrome (MetSyn), body fat percentage and cholesterol measurements.

**Conclusion:**

A high proportion of overweight and obese male career firefighters underestimate their measured BMI categories. As a result, they are unlikely to fully appreciate the negative health consequences of their excess weight. The results of this study emphasize the importance of objectively measuring BMI and then informing patients of their actual weight status and the associated disease risks.

## Background

According to the World Health Organization (WHO), the prevalence of obesity in the US has reached 44% in males between 30 and 100 years of age [1]. Higher Body Mass Index (BMI) has been associated with increased risk of cardiovascular disease (CVD) and certain types of cancer [2,3]. On the other hand, weight loss has been shown to decrease the risks associated with excess weight and obesity. Even a small amount of weight loss has positive effects on cardiovascular risk factors [4].

Perception of one’s weight status as non-ideal is usually the basis for any subsequent decision to change one’s weight. Accordingly, there is a strong association between perception of weight and effective weight control in adults [5]. Therefore, accurate awareness of weight status is an increasingly important adjunct to achieving successful weight loss. Unfortunately, recent evidence emphasizes that the rapid societal increase in overweight and obesity has been accompanied by fewer overweight people correctly perceiving themselves as belonging to the overweight category [6]. One recent study found that two-thirds of already obese individuals did not recognize their obesity. Moreover, despite their already excessive weights they believed they were at low risk of developing obesity [7].

In parallel, recent research has found that obesity in the United States (US) fire service has reached epidemic levels [8-11]. In a prospective evaluation, obesity prevalence in this occupation increased significantly over time [8]. Moreover, a recent population-based investigation of career and volunteer firefighters used BMI, waist circumference and body fat measures to validate prevalence estimates done using BMI with the latter measures of abdominal obesity and adiposity (fat mass) [10]. This investigation proved that the high obesity prevalence found among firefighters using BMI was not due to misclassification of increased muscle mass as adiposity (fat mass), which was contrary to popular belief in the fire service. In fact, obesity was even more prevalent when assessed by body fat rather than BMI, while the misclassification of muscular firefighters as obese by BMI was infrequent [10]. Furthermore we have described that obesity defined using BMI measures is associated with CVD risk factor clustering [8,9], lower cardio-respiratory fitness (CRF) [9,12,13] as well as higher risk of job disability in firefighters [14].

In agreement with the above results, several general population studies have recently reported an increase in overweight and obesity over time. All but one [15] also described a parallel increase in misperceptions of excess weight, expressed as failing to recognize oneself as being overweight or obese [6,16,17].

Recently, body weight misperception has been shown to be associated with gender, health beliefs, ethnicity and socioeconomic status [5,7,18,19]. However, to the best of our knowledge, no study has previously described weight perception and its association with cardiovascular risk factors, CRF and body composition in firefighters or even in the general population per se. The purposes of this study were to describe and quantify the discrepancy between weight perception and actual BMI in a high risk public safety profession; and secondarily, to compare CVD risk factors across categories of measured BMI and perceived weight status.

## Methods

### Study population

Male career firefighters, 18 years of age and older, were recruited from fire departments in two Midwestern states. Inclusion criteria were completing a maximal exercise test during the course of a fire department- sponsored medical examination, and working full duty without any work restrictions at the time of the examination. Excluded subjects failed to meet one or more of the above criteria or had undergone the index exercise tests for the evaluation of symptoms, retirement pensions, disability and/or exit examinations. The study was approved by the IRB of Harvard School of Public Health and local IRBs as appropriate. All participants signed an informed consent.

### Assessment of cardiovascular risk factors

Height was measured in the standing position with a clinic stadiometer. Body weight was measured with bare feet and in light clothes on a calibrated scale. BMI was calculated as the weight in kilograms divided by the square of height in meters. Body fat percentage (body fat%) was either estimated by the use of a Bioelectrical Impedance Analyzer (BIA) or with skin fold measures depending on the protocol of the respective fire department. Blood pressure was measured using an appropriately sized cuff with the subject in the seated position. Resting pulse and blood pressure were obtained from the physical examination. Fasting venous blood samples were analyzed for total cholesterol (total chol), HDL-cholesterol (HDL-chol), LDL-cholesterol (LDL-chol), the ratio between total- and HDL chol (Tchol/HDL), c- reactive protein and glucose using standardized methods.

### Cardio-respiratory fitness

CRF was measured using symptom- limited maximal treadmill exercise testing with ECG monitoring and estimation of oxygen consumption (metabolic equivalents (METS)) according to the Bruce protocol [20]. The participants were encouraged to continue exercise until volitional exhaustion; even after exceeding 85% of their maximum predicted heart rate (maximal predicted heart rate is defined as 220 minus age). The cohort achieved an average of 97.9% (SD 6.6) of maximal age-predicted heart rate on these tests. During the exercise test CRF was determined from the maximum or peak METS achieved at peak exercise. Heart rate recovery at one minute (HHR1) was calculated as peak heart rate minus heart rate at one minute into recovery following the test.

### Definition of the metabolic syndrome (MetSyn)

The prevalence of MetSyn among the study population was determined using modified criteria from the Joint Scientific Statement [21,22]. If three or more of the following five risk factors were present then the participant was categorized as having MetSyn: abdominal obesity- modified here to BMI ≥30; hypertriglyceridemia (≥150 mg/dL); reduced HDL-chol <40 mg/dL; elevated blood pressure (systolic ≥130 and/or diastolic ≥85 mmHg) and/or antihypertensive drug treatment; or hyperglycemia (blood glucose ≥100 mg/dL) [21].

### Assessment of weight perception and weekly exercise

Self-reports of weight perception and weekly exercise were extracted from responses to a health and lifestyle questionnaire as previously described from our group [12]. Consented study participants were given standardized written instructions for completing the multiple choice survey regarding eating, health, exercise, sleep, and work habits as honestly and as best as they could. They were also informed that the completed questionnaires would be confidential and would not become part of their fire department or medical record. To assess weekly exercise, the following question was asked: “Most weeks, I exercise .... (include home/work/gym & elsewhere)” [possible answers: one day or less, 2–4 days, 5 days or more]. To assess weight perception, answers to the following questions were selected. (1) “I think my body weight is… “[possible answers: underweight (skinny); healthy/normal or muscular; overweight; obese (fat)]. (2) “In the next year, I want my body weight to go … “[possible answer: Down a lot (>10 pounds); Down a little (5–10 pounds); Not changed (< 5 pounds); Up a little (5–10 pounds); Up a lot (>10 pounds)].

The combination of objectively measured BMI groups and the *self-perceived weight* categories gave rise to 12 groups (BMI category/*self-perception weight category*)*.* The following groups were not included in the analyses due to very small numbers: [overweight BMI/*underweight (skinny)*; obese BMI/*underweight (skinny)*; normal BMI/*obese* (n = 0)] and [normal BMI/*underweight (skinny)*; normal BMI/*overweight*; overweight BMI/*obese (fat)* (n ≤ 9)].

### Statistical analysis

Baseline characteristics were described using the mean (SD) in the case of quantitative variables and the frequency in case of categorical variables. Group comparisons were calculated using ANOVA. Linear regression models were used to evaluate the associations among the six BMI/weight perception categories (independent variable) and various cardiovascular risk factors (dependent variables) while adjusting for other co-variables. Logistic regression analyses were used to calculate odds ratios (95% CI) for underestimating one’s weight category (dependent variable) as a function of BMI as a continuous independent variable. Analyses were performed using SAS 9.2 (SAS Institute Inc., Cary, NC, USA). All tests presented are two-sided and a p-value <0.05 is considered significant.

## Results

**Figure 1:**
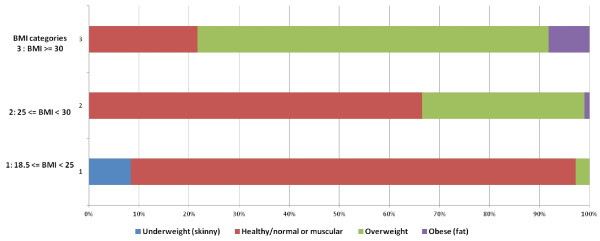
**Self perception of weight compared with the calculated BMI.** Colored bars represent different weight perception groups.

**Figure 2:**
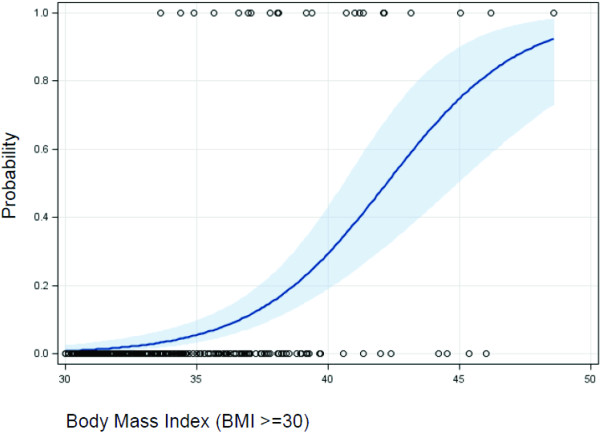
Prediction probabilities and 95% prediction limits of correctly perceiving weight group as a function of BMI among obese participants.

The cohort’s (n = 768) baseline characteristics are summarized in Table 1. The mean BMI and body fat were both close to respective obesity cutoff criteria: 29.4 (4.4) versus 30 kg/m^2^ and 23.6% versus 25%. The prevalence of underestimating one’s weight category furthermore increased with age (p-value <0.0001). Figure 1 illustrates firefighters’ perceptions of their weight within each measured BMI category. In the measured normal BMI category (≥18.5 and <25), 89% of the study participants perceived themselves correctly as “healthy/normal or muscular”. In the overweight BMI category (25 ≤ BMI < 30) only 32.4% rated themselves correctly as overweight. Furthermore in the obese BMI category (BMI ≥ 30) only 8.2% of firefighters perceived themselves correctly as obese (Figure 1). Moreover, with every one unit increment in the BMI, the odds of underestimating one’s BMI measured category was increased by 24% [OR = 1.241 (95% CI 1.176–1.308)] after adjustment for age and CRF (Table 2). However, if we examined only obese subjects (BMI ≥ 30), as obesity increased, the probability of correctly perceiving one’s measured weight category actually increased as BMI increased (Figure 2).

**Table 1:** Baseline characteristics (n = 768)

Age *mean (SD)*	37.6 (8.5)
BMI *mean (SD)*	29.4 (4.4)
CRF (maximal METS) *mean (SD)*	12.8 (1.6)
Body fat% *mean (SD)*	23.6 (6.6) in n = 232
Blood glucose *mean (SD)*	94.0 (21.0)
Resting systolic BP *mean (SD)*	122.6 (12.6)
Resting diastolic BP *mean (SD)*	79.9 (8.0)
Resting heart rate *mean (SD)*	69.5 (11.4)
HDL-chol *mean (SD)*	45.3 (11.2)
LDL-chol *mean (SD)*	120.7 (34.1)
Total- chol *mean (SD)*	195.2 (38.7)
TChol/HDL ratio mean (SD)	4.6 (1.5)
Heart rate recovery at one minute *mean (SD)*	33.1 (13.5)
c-reactive protein *mean (SD)*	2.2 (3.7) in n = 389

**Table 2:** Odds Ratios of underestimating one’s BMI category as a function of increasing BMI (continuous)

	**Model 1**	**Model 2**	**Model 3**
OR (95% CI)	1.201 (1.146-1.258)	1.210 (1.154-1.269)	1.241 (1.176-1.308)

Model 1: unadjusted; Model 2: adjusted for age; Model 3: adjusted for age and CRF (maximal METS).

*BMI* (Body Mass Index), *CRF* (Cardio-Respiratory Fitness), *METS* (Metabolic Equivalent), *OR* (Odds Ratio), *CI* (Confidence Interval).

Cardiovascular risk factors are described and compared among the six different BMI/*weight perception* groups in Table 3. With and without adjustment for age, differences were statistically significant among the groups for CRF, amount of weekly exercise, MetSyn prevalence, resting systolic and diastolic blood pressure, HDL-, LDL- and total chol as well as for the ratio between total- and HDL chol, body fat%, c- reactive protein and glucose (Table 3). If additionally adjusted for BMI, the differences remained statistically significant for CRF, amount of weekly exercise, MetSyn, HDL- LDL- and total chol as well as Tchol/HDL and body fat%.

**Table 3:** Cardiovascular Risk Factors as a function of Body Mass Index and weight perception categories

**n**	**95**	**263**	**128**	**61**	**198**	**23**	**p-value**^**2**^	**p-value**^**3**^	**p-value**^**4**^
BMI category	normal	overweight	overweight	obese	obese	obese	n/a	n/a	n/a
*Self reported weight category*^*1*^	*healthy/nl*	*healthy/nl*	*overweight*	*healthy/nl*	*overweight*	*obese*	n/a	n/a	n/a
weight perception	correct	underestim	correct	underestim	underestim	correct	n/a	n/a	n/a
Age mean (SD)	35.9 (9.9)	36.0 (8.2)	39.4 (8.5)	36.4 (7.8)	39.3 (8.0)	39.3 (6.9)	**<0.0001**	n/a	n/a
BMI mean (SD)	23.7 (1.0)	27.2 (1.3)	28.3 (1.4)	32.5 (2.3)	33.8 (3.1)	39.6 (3.8)	**<0.0001**	**<0.0001**	n/a
CRF (maximal METS) mean (SD)	13.7 (1.6)	13.4 (1.2)	12.6 (1.3)	12.6 (1.3)	11.8 (1.6)	10.8 (1.6)	**<0.0001**	**<0.0001**	**0.0052**
Weekly exercise (days/week) (one day or less) n (%)	15 (16.0)	18 (6.9)	30 (23.4)	5 (8.2)	44 (22.5)	10 (43.5)	**<0.0001**	**0.0010**	**<0.0001**
Weekly exercise (2-4 days) n (%)	53 (56.4)	157 (60.2)	84 (65.6)	36 (59.0)	130 (66.3)	13 (56.5)			
Weekly exercise (5 days or more) n (%)	26 (27.7)	86 (33.0)	14 (10.9)	20 (32.8)	22 (11.2)	0 (0.0)			
MetSyn n(%)	4 (4.2)	20 (7.6)	20 (15.6)	33 (54.1)	118 (59.6)	18 (78.3)	**<0.0001**	**<0.0001**	**<0.0001**
HRR1 mean (SD)	36.5 (14.4)	33.8 (14.0)	31.3 (13.7)	32.3 (11.7)	32.2 (12.9)	30.5 (10.8)	0.0702	0.2069	0.5954
RSBP mean (SD)	118.8 (12.3)	121.1 (12.0)	121.4 (12.4)	125.6 (14.6)	125.8 (12.5)	125.5 (10.4)	**<0.0001**	**<0.0001**	0.6621
RDBP mean (SD)	76.7 (8.0)	78.3 (7.6)	79.5 (7.4)	82.0 (8.2)	83.0 (7.8)	81.5 (5.6)	**<0.0001**	**<0.0001**	0.1229
HDL- chol mean (SD)	50.4 (11.0)	48.7 (11.3)	43.2 (9.6)	43.3 (10.0)	40.8 (10.1)	40.8 (11.4)	**<0.0001**	**<0.0001**	**0.0004**
LDL- chol mean (SD)	109.4 (34.0)	117.7 (34.0)	129.3 (33.8)	116.8 (25.1)	125.9 (34.3)	121.8 (41.5)	**<0.0001**	**0.0023**	**0.0054**
TChol mean (SD)	181.7 (37.1)	191.9 (38.1)	203.7 (43.6)	191.0 (29.1)	200.3 (36.2)	211.0 (46.0)	**<0.0001**	**0.0010**	**0.0020**
TChol/HDL ratio mean (SD)	3.8 (1.3)	4.2 (1.4)	4.9 (1.5)	4.6 (1.3)	5.1 (1.4)	5.5 (1.7)	**<0.0001**	**<0.0001**	**0.0002**
Body fat(%) mean (SD)	16.0 (6.8) n = 32	19.8 (4.0) n = 72	23.8 (3.4) n = 36	27.4 (4.4) n = 20	29.1 (3.6) n = 64	32.5 (4.4) n = 8	**<0.0001**	**<0.0001**	**0.0055**
c-reactive protein mean (SD)	1.5 (2.6) n = 58	1.8 (3.9) n = 136	2.4 (6.0) n = 48	2.0 (2.3) n = 35	3.1 (2.6) n = 102	3.4 (3.6) n = 10	**0.0331**	**0.0336**	0.7612
Blood glucose mean (SD)	87.2 (12.2)	92.4 (20.8)	94.8 (13.7)	94.8 (21.8)	97.7 (26.9)	101.7 (19.1)	**0.0009**	**0.0105**	0.8976

^1^Healthy/normal or muscular; Overweight; Obese (fat).

^2^crude; ^3^ age adjusted; ^4^ adjusted for age and BMI.

*BMI* (Body Mass Index), *CRF* (Cardio-Respiratory Fitness), *METS* (Metabolic Equivalent), *MetSyn* (Metabolic Syndrome), *HRR1* (Heart Rate Recovery at one minute into recovery), *RSBP* (Resting Systolic Blood Pressure), *RDBP* (Resting Diastolic Blood Pressure), *TChol/HDL* (Ratio between total cholesterol and HDL).

Among the three groups where measured BMI category is normal or overweight, the following parameters were significantly different: CRF, amount of weekly exercise, MetSyn, HRR1, HDL-, LDL- and total chol, as well as the Tchol/HDL, body fat% and blood glucose. After adjustment for age and BMI the following parameters were significantly different: CRF, amount of weekly exercise, HDL- and total chol, as well as the Tchol/HDL and body fat%.

In comparison when analyses were limited to only those groups where measured BMI was in the obese category (but weight perception was normal, overweight or obese), the analysis showed significant differences for CRF, body fat%, Tchol/HDL and HDL. After adjustment for age and BMI, only the difference for total chol remained significant.

Additionally the three groups of obese participants answered differently when asked about desired weight change in the next year based on their self-perceptions of their weight status. In the group that perceived themselves of normal weight 34.4% wished to reduce their weight more than 10 pounds compared to 69% among those perceiving themselves as overweight and 82.6% in the group that perceived themselves correctly as obese (p-value overall <0.0001).

## Discussion

We found that a high proportion of overweight and obese male career firefighters underestimate their weight group (68%). This is consistent with previously reported findings in the literature [23]. We also found the risk of underestimating one’s weight category increased by 24% with each additional unit of BMI after adjustment for age and CRF. These findings are also consistent with other studies [6,16,17].

Novel findings from this study relate to cardiovascular risk factors as a function of weight perception. We found that risk parameters varied significantly not only with measured BMI, but also with weight perception. Among those who are overweight or obese, CVD risk showed worsening trends as the BMI increased and weight perception became more accurate. This finding seems to be explained by further increases in BMI and body fat that are found among overweight and obese persons who correctly perceive themselves as overweight or obese, rather than normal or muscular. In fact, obese firefighters who perceived themselves as obese were close to morbidly obese on average (BMI > 39). An alternate view (and not mutually exclusive one) is that overweight and obese subjects who view themselves as “normal/healthy or muscular” are actually more active, have higher CRF and better CVD risk profiles than obese subjects who perceive themselves as heavier. However, in the present study, while this was true, overweight and obese participants who perceive themselves as normal/muscular still have worse CVD risk parameters than colleagues whose measured BMI is normal. Thus, any differences based on perception appear to be accounted for by differences in the degree of adiposity as measured by BMI and body fat.

Correctly perceiving one’s BMI category does not only imply knowing one’s weight relative to their height. It also seems to be dependent on social standards and peer groups. If most of the peers one compares himself to are overweight or obese, there is a danger of perceiving overweight as “normal” or “standard size” [23]. In our cohort 86.1% of firefighters were overweight and obese, so it is quite likely that shifts in standards of weight perceptions are occurring in the fire service. During the past decade the desired weight of Americans has increased alongside the increase of obesity. This is suggestive that at some point the assessment of body weight is made in relation to distribution of weight in the general population [24].

An additional interesting finding regarded risk factor clustering. Our study showed that the MetSyn prevalence was highly dependent on both measured and perceived weight categories. This was especially true in those with a BMI measured in the obese category. The prevalence of MetSyn was quite high among obese firefighters and increased from 54.1% (self-perceived normal/healthy/muscular) up to 78.3% (self-perceived obese). Therefore our data may suggest that especially if subjects are obese but relatively healthy they may be more likely to underestimate their weight category as well as their weight-related health risks. Recognizing this might provide an opportunity for increasing and emphasizing education concerning healthy weight and weight maintenance strategies [25]. While the less obese subjects who perceive themselves as healthy are in fact healthier than even heavier co-workers, they are clearly much less healthy than leaner co-workers on multiple objective measures.

### Strengths of the present study

A particular strength of the present study was the large sample size which allowed for adequate power and adjustment for confounders. BMI was calculated from measured weight and height in bare feet and light clothes during medical examinations which avoided self-reporting biases towards lower weights and taller heights and other random misclassification. We also were able to compare the associations of perceived weight category on objectively measured markers of CVD risk, which permitted us to characterize the health status of the various subgroups. Moreover, our study distinguished between overweight and obese. When most overweight or obese people perceive themselves as overweight, they fail to appreciate the extent of how far they are from their healthy weights [5]. Finally because of the very homogenous study group there was no need to adjust for socioeconomic status (education, income, occupation).

### Limitations of the present study

Our study does have some modest limitations, including the cross-sectional design. Because of previously described gender differences in weight perception and the very small number of female career firefighters, we limited our investigation to male firefighters. Furthermore, although we had very complete data overall, in the case of body fat% and c-reactive protein measurements, some firefighters were examined before these items were added to their departments’ medical exam protocols. Therefore, we lacked complete data for these two variables. Finally, with regard to body composition and MetSyn determination, we lacked measures of waist circumference. However, BMI has been shown to be accurate measure of adiposity in firefighters [10].

## Conclusion

We found that a high proportion of overweight and obese male career firefighters underestimate their measured BMI categories. In fact, the risk of underestimating one’s BMI category increased by 24% with each additional unit of measured BMI after adjustment for age and CRF. Furthermore when divided into six groups based on combination of measured BMI category and weight self-perception, there were significant differences among the groups for most cardiovascular risk factors which remained statistically significant for CRF, amount of weekly exercise, prevalence of MetSyn, body fat percentage and cholesterol measurements after adjustment for age and BMI.

The results of this study emphasize the importance of objectively determining BMI and then informing patients of their actual weight status and the associated disease risks. This is of particular importance in public safety occupational settings like firefighting where the public depends on workers to be healthy and fit for duty.

## Competing interests

The other authors report no conflict of interest.

## Authors’ contributions

DMB contributed to the study design, performed statistical analyses, wrote and edited the manuscript; CAC performed statistical analyses and edited manuscript; AJT contributed to the study design, drafted and edited manuscript; SAJ participated in study recruitment and design and edited manuscript; SNK planned and designed the study and edited manuscript. All authors read and approved the final manuscript.

## Pre-publication history

The pre-publication history for this paper can be accessed here:

http://www.biomedcentral.com/1471-2458/12/480/prepub
